# Microplastics in Greylag Goose (*Anser anser*) Feces from Lake Erçek (Eastern Anatolia, Türkiye): Occurrence, Temporal Variation, and Polymer Characterization

**DOI:** 10.3390/toxics14020108

**Published:** 2026-01-23

**Authors:** Emrah Celik

**Affiliations:** 1 Vocational School of Technical Sciences, Department of Forestry, Hunting and Wildlife Program, Igdir University, 76000 Igdir, Türkiye; celikemrah822@gmail.com; Tel.: +90-553-477-7982; 2 Ornithology Research and Application Centre (ORNITHOCEN), Igdir University, 76000 Igdir, Türkiye

**Keywords:** waterfowl, non-invasive monitoring, freshwater contamination, inland waters, birds

## Abstract

**Background:** Microplastics (MPs; <5 mm) are pervasive contaminants that can compromise freshwater wetland integrity and wildlife health, yet field evidence from inland systems and non-invasive biomonitoring remains limited. To address this gap, we provide a non-invasive, feces-based baseline for a key wintering waterbird in an inland soda-lake wetland of Türkiye, supported by polymer confirmation. **Methods:** We evaluated MP occurrence in fecal deposits of the Greylag Goose (*Anser anser*), a key wintering waterbird at Lake Erçek (Eastern Anatolia, Türkiye), using non-invasive sampling across five periods (October 2024–February 2025). We collected 400 fecal deposits and pooled them into five time-specific composite samples. Accordingly, temporal comparisons are presented descriptively at the composite (period) level rather than as individual-level statistical inference and quantified suspected MPs by type, shape, size, and color; a representative subset (>300 µm; ~20%) was polymer-confirmed by FT-IR, and particle surfaces were examined by SEM–EDX. **Results:** In total, 959 suspected MP items were recovered, corresponding to an estimated 1.75–2.85 items per fecal deposit (composite-derived; mean 2.40). MP counts peaked in late autumn–early winter (Time 2–Time 3) and declined toward late winter (Time 5). Fibers predominated (37.22%), followed by fragments (30.55%) and pellets (18.77%); the most frequent size class was 100–300 µm (30.25%), and white/transparent particles were most common (38.52%). FT-IR identified polystyrene, polyethylene, and polyvinyl chloride, while SEM–EDX indicated weathered polymeric surfaces. **Conclusions:** These findings provide baseline evidence of MP exposure in an inland wetland waterbird and support feces-based monitoring for comparative assessments.

## 1. Introduction

Plastic production and consumption have generated persistent waste streams that fragment into microplastics (MPs, <5 mm), creating a pervasive contaminant of global concern [1,2,3,4]. Microplastics can be transported through aquatic systems, redistributed by resuspension and wind, and persist in sediments, thereby increasing long-term exposure opportunities for wildlife [5,6,7,8,9,10]. Wetlands are particularly vulnerable because they receive inputs from agriculture, settlements, runoff, and waste disposal, and can function as sinks where MPs accumulate [11]. Evidence from intertidal wetlands shows that plastics and microfibers occur simultaneously in sediments, macroinvertebrates, and birds, supporting the role of wetland food webs in transferring MPs to avifauna [11,12,13]. Birds are valuable sentinels for MP contamination because they integrate exposure across habitats and diets and can reflect both local and broader landscape-scale pollution patterns [14,15,16]. Microplastics have been documented in multiple avian matrices, and fecal-based monitoring is increasingly used as an ethical and scalable alternative to invasive approaches [17]. Beyond ingestion, accumulating evidences indicate that inhalation of airborne microplastics and micro(nano)plastics may contribute materially to avian exposure, including demonstrable contamination in lung tissues, which in turn strengthens the case for rigorous particle-level and polymer-specific characterization in avian monitoring studies [18,19,20,21]. However, a substantial portion of the avian microplastic literature still relies on necropsy-based or opportunistic sampling of dead individuals, an approach that can introduce representativeness biases (e.g., stranded or otherwise compromised birds) and inherently restrict repeated temporal surveillance compared with non-invasive feces-based monitoring [17,18,22].

Non-invasive sampling of feces (or guano) provides a practical pathway to monitor live populations repeatedly and to assess MPs in protected or hard-to-sample species [17,18,23,24,25,26]. Fecal-based MP assessments have been applied successfully across diverse taxa and environments, including urban waterbirds, shorebirds, and protected-area bird communities [11,17,24]. In a large protected wetland reserve, MPs were detected in the guano of multiple bird species, and abundance differed among ecological groups, indicating that habitat use and feeding ecology can modulate exposure [24]. In urban freshwater systems, fecal sampling during ringing/banding revealed high MP burdens in waterbirds, highlighting urban waterbodies as potential hotspots of exposure [17]. Shorebird-focused wetland work further supports that MPs present in sediments and invertebrates can be reflected in birds, strengthening the rationale for integrating fecal monitoring with environmental matrices [11]. Across avian feces and guano studies, fibers often dominate the recovered particles, although the proportion varies substantially among habitats, taxa, and analytical thresholds [24]. Methodological factors—including minimum size cut-offs, partial spectroscopic confirmation, and contamination control—strongly influence reported MP metrics and comparability among studies [25]. For example, a seabird feces study implemented a 100 µm lower cut-off and used partial filter-area Raman verification alongside blanks, illustrating common feasibility constraints and Quality Assurance (QA)/Quality Control (QC) expectations in fecal workflows [25]. Seasonal variability in MP occurrence in bird droppings has also been reported in wetland settings, suggesting that time-structured sampling can reveal temporal signals linked to hydrology and human activity [27]. Despite the expanding global literature, inland wetlands of Türkiye remain underrepresented in feces-based MP monitoring, limiting baseline data for conservation and management in key migratory landscapes [14]. The Greylag Goose (*Anser anser*) is a suitable biomonitor candidate because it is abundant, forms large flocks, uses shallow wetlands and adjacent agricultural areas, and may ingest MPs incidentally while grazing and interacting with sediment-influenced habitats [24]. Although the number of studies linking microplastics and birds has increased rapidly, many reports are primarily species-specific occurrence evidence and/or rely on invasive sampling (e.g., gastrointestinal contents). In contrast, feces-based sampling offers a non-invasive and repeatable monitoring approach that is increasingly applied to quantify microplastic exposure in birds and to enable spatio-temporal surveillance across habitats. Recent fecal studies demonstrate the utility of this approach across freshwater and urban settings [17,28], and also in waterfowl [29].

Within the Lake Van closed basin, microplastic contamination has been reported in adjacent surface waters and aquatic environments [30,31], yet information on exposure of free-living waterbirds remains limited. Therefore, the present study provides a polymer-confirmed baseline of microplastics in non-invasively collected Greylag Goose (*Anser anser*) feces from the Lake Erçek basin.

Here, we provide the first evidence on MP occurrence and characteristics in fecal samples of Greylag Goose from Lake Erçek (Van, Eastern Anatolia, Türkiye), using visual identification supported by FT-IR and SEM-based characterization. Specifically, we aimed to (i) quantify MP abundance across five temporal sampling periods, (ii) describe particle size, type, and color distributions, and (iii) determine polymer composition of representative particles to infer potential sources. We expected that MPs would be detectable in all sampling periods, reflecting persistent environmental inputs to the lake system. We further expected that fibers would represent the dominant particle type and that polymer composition would be consistent with commonly reported consumer and packaging plastics in feces-based avian studies. Finally, we expected that MP abundance would vary among periods due to seasonal shifts in wetland dynamics and anthropogenic pressure, as suggested by seasonal signals reported in wetland bird-dropping surveys.

## 2. Materials and Methods

### 2.1. Study Area

**Figure 1:**
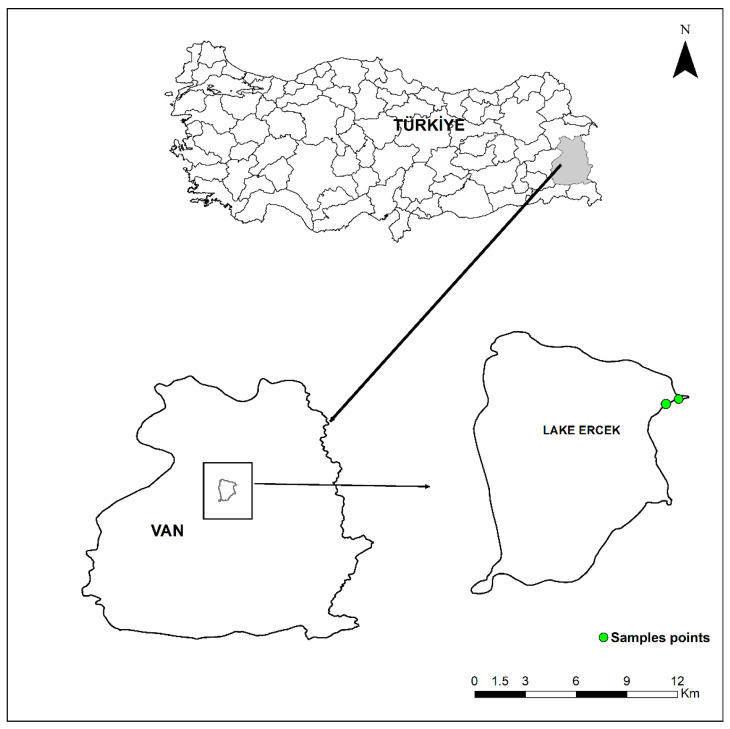
Location of the study area and sampling stations.

Lake Erçek (Van, Türkiye) is a tectonic, endorheic (closed-basin) soda lake located in the Van Lake Basin, Eastern Anatolia, Türkiye (1803 m a.s.l.; surface area 106.2 km^2^; mean depth 18.45 m) [32]. The lake functions as an important wintering and staging habitat for large waterbird assemblages, including the Greylag Goose (*Anser anser*). Field sampling was conducted during five periods: 5–20 October 2024 (Time 1), 10–24 November 2024 (Time 2), 1–14 December 2024 (Time 3), 10–20 January 2025 (Time 4), and 5–15 February 2025 (Time 5). Sampling locations were selected in shoreline recreation areas and adjacent agricultural mosaics commonly used by geese for roosting and foraging, to facilitate reliable feces attribution to the target species while capturing heterogeneous anthropogenic pressures (Figure 1).

### 2.2. Sampling Strategy and Feces Collection

**Figure 2:**
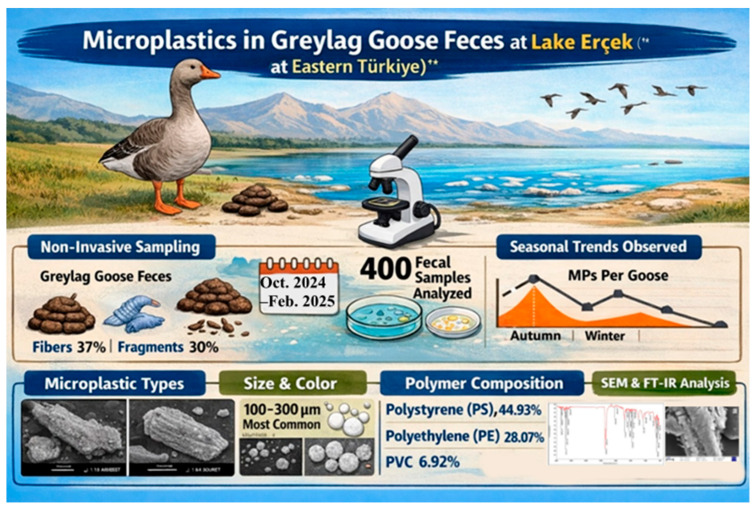
Workflow of the study (the author discloses that the figure was composed based on the content of the present study using the aid of artificial intelligence).

Fresh fecal deposits of Greylag Goose were collected from multiple localities representing different land-cover contexts and anthropogenic influence [18,24]. Prior to sampling, sites were surveyed, and geographic coordinates were recorded [23,33]. To reduce the likelihood of mixed-species attribution, sampling avoided areas where multiple bird species were densely aggregated [17,18]. After flocks departed a resting area, feces were collected following a 30–45 min waiting period [23]. Samples were collected as aseptically as feasible without stratification by sex or age [33]. Fresh fecal deposits were collected immediately after deposition and transferred using a stainless-steel spatula into pre-cleaned 50 mL glass bottles (rinsed with ultrapure water). To minimize soil inclusion, only intact deposits with a clearly visible boundary from the substrate were sampled. When droppings were on soft ground, only the upper portion was collected, and the feces–substrate interface was avoided. Bottles were closed promptly using aluminum-foil wrapping/lining to minimize contact with plastic components [34], transported in cooled containers, and stored at −20 °C until analysis [35]. The workflow is as follows Figure 2.

### 2.3. Microplastics (MPs) Isolation

Frozen fecal samples were thawed at room temperature. For each sampling period, the deposits were combined into a single time-specific composite in a pre-cleaned 800 mL glass beaker using instruments rinsed with ultra-pure water, resulting in five homogenized composite samples. For each period, 80 fecal deposits were pooled. Abundance was expressed as items per fecal deposit (composite-derived) by dividing the total number of suspected MP items recovered from each composite by the number of deposits pooled. We did not normalize counts by dry mass because individual deposit masses were not recorded. The composites were dried at 40 °C for 48 h to remove moisture. The dried material was digested with 10% potassium hydroxide (KOH) at 50 °C for 48 h [35,36]. Floating material was removed, and vacuum filtration was performed using Whatman GF/A filters (47 mm diameter, 1.6 µm pore size). The remaining residue was further oxidized with 30% hydrogen peroxide (H_2_O_2_) until organic matter was visibly reduced, and the digest was filtered again using the same type of filter. The filters were stored in closed glass Petri dishes (60 mm) until examination. To minimize contamination, all wet-lab steps were conducted using glass/metal equipment pre-rinsed with filtered ultrapure water [37], samples and filters were kept covered whenever possible, and working surfaces were cleaned prior to processing [37,38]. Operators wore non-synthetic laboratory coats and gloves [33]. However, quantitative field and procedural blanks were not performed, so we could not correct the results for airborne contamination (particularly fibers). This limitation is acknowledged explicitly (Section 5).

The filters were examined under a stereomicroscope to visually identify suspicious MPs. The particles were counted and photographed, and measurements (length and width) were obtained using ImageJ.JS. To reduce selection bias, each filter was scanned systematically across the entire surface (grid-like transects), and all particles meeting predefined visual MP criteria were recorded prior to spectroscopic confirmation. Classification was performed for MPs based on standard visual criteria [39], and particles were classified by type (fiber, fragment, pellet, film, foam), shape (irregular, elongated, spherical, line, flat), size class (<100 µm, 100–300 µm, 300–500 µm, 500–1000 µm, >1000 µm), and color (white/transparent, black, gray, brown, blue, yellow, orange, red/pink, green) [18]. After the certification process, particles > 300 µm in size were recovered from the filters under a microscope using a fine needle and stored in sterile glass bottles for polymer and surface characterization.

### 2.4. SEM–EDX Sample Processing and Analysis

Due to handling constraints, only particles > 300 µm were analyzed by SEM–EDX. Selected MPs were placed onto carbon tape mounted on metal stubs and sputter-coated with a thin gold layer (Quorum SC7620) prior to imaging. Because specimens were Au sputter-coated, Au-related characteristic peaks (notably in the ~2.1 keV region) can appear in EDX spectra and were not interpreted as intrinsic particle composition. Particles were imaged using a TESCAN Vega 3 SEM at 5.0 kV across 85×–2840× magnification [40]. For EDX, two points per particle were targeted to acquire representative spectra and evaluate dominant elemental signals consistent with polymeric material.

### 2.5. Fourier-Transform Infrared (FT-IR) Analysis

Polymer identification was performed using Fourier-transform infrared (FT-IR) spectroscopy. Because ATR-FTIR requires manual positioning of particles and the effective beam size can exceed smaller particle dimensions, spectral acquisition for <300 µm items is prone to particle loss, mixed spectra, and reduced match confidence. Accordingly, only selected MPs > 300 µm were analyzed, representing ~20% of recovered items. Therefore, polymer identification is restricted to this size-selected subset and cannot be extrapolated to the dominant < 300 µm fraction; polymer composition is reported as indicative rather than exhaustive. Recent methodological discussions emphasize size-dependent confirmation bias and the need for harmonized QA/QC in biological matrices [41]. FT-IR spectra were recorded using a JASCO FT/IR-6600 over 400–4000 cm^−1^ at 1 cm^−1^ resolution. Polymer assignment was based on spectral matching against reference libraries and standard (Aldrich^TM^ Organometallic, Inorganic Spectral Library, Van Yuzuncu Yıl University, Van, Türkiye, Thermo Scientific).

### 2.6. Data Treatment and Descriptive Analysis

Because fecal deposits were pooled into a single composite per sampling period (n = 5 composites), the composite (period) is the only independent analytical unit. Therefore, results are reported descriptively (counts, percentages, and period-level profiles) and figures are used to visualize temporal patterns. No inferential hypothesis tests (e.g., ANOVA or post hoc multiple comparison tests) are presented for particle type, shape, size class, or color. To explore potential co-variation among particle attributes (type, shape, size class, and color) across sampling periods, we computed Pearson correlation coefficients using period-level category counts and visualized them as correlation heatmaps (Supplementary Figure S1). Because the number of independent composites is small (n = 5), this analysis is intended as an exploratory descriptive summary and is interpreted qualitatively.

## 3. Results

### 3.1. Abundance and Temporal Variation

Fecal samples of Greylag Goose collected from Lake Erçek across five sampling periods confirmed contamination by microplastics (MPs; <5 mm). In total, 959 MP items were recovered from 400 fecal deposits pooled into five time-specific composite samples. corresponding to an estimated 1.75–2.85 items per fecal deposit (composite-derived), with an overall mean of 2.40 items per fecal deposit (composite-derived). Total counts increased from October to December (Time 1 to Time 3) and declined in January and February (Time 4 to Time 5) (Table 1).

### 3.2. Size Distribution

**Figure 3:**
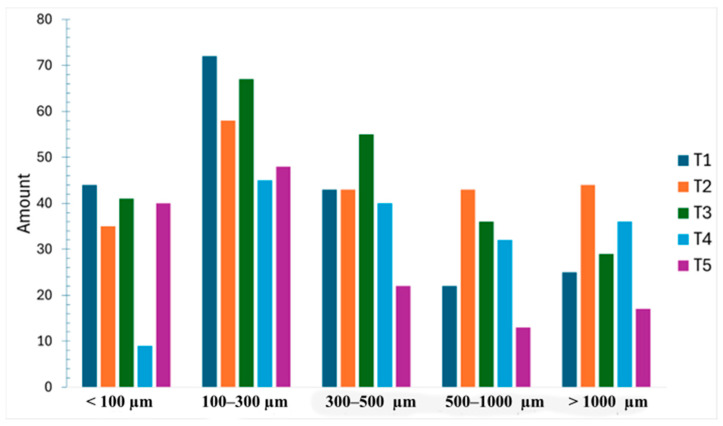
Microplastics by size class across sampling periods (n = 5 composite samples).

Particles were classified into five size classes: <100 µm, 100–300 µm, 300–500 µm, 500–1000 µm, and >1000 µm. The most frequent class was 100–300 µm (n = 290; 30.25%), followed by 300–500 µm (n = 203; 21.17%), <100 µm (n = 169; 17.62%), >1000 µm (n = 151; 15.74%), and 500–1000 µm (n = 146; 15.22%). Across periods, the 100–300 µm class remained the most frequent, followed by 300–500 µm and larger fractions (Figure 3).

### 3.3. MPs Type

**Figure 4:**
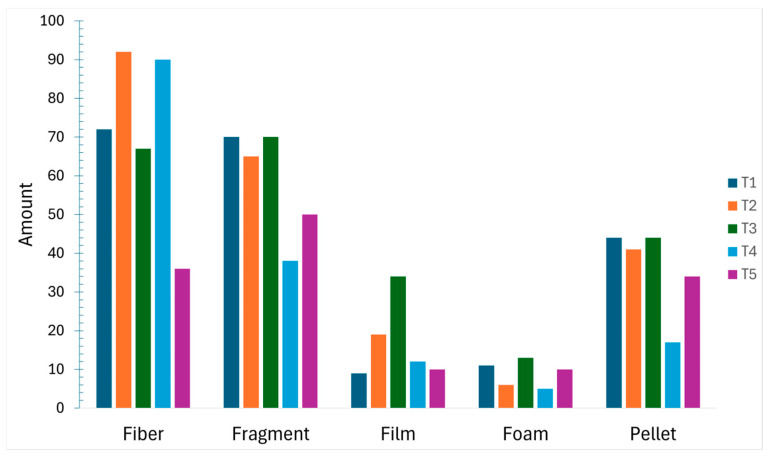
Particle-type composition of microplastics (n = 959).

**Figure 5:**
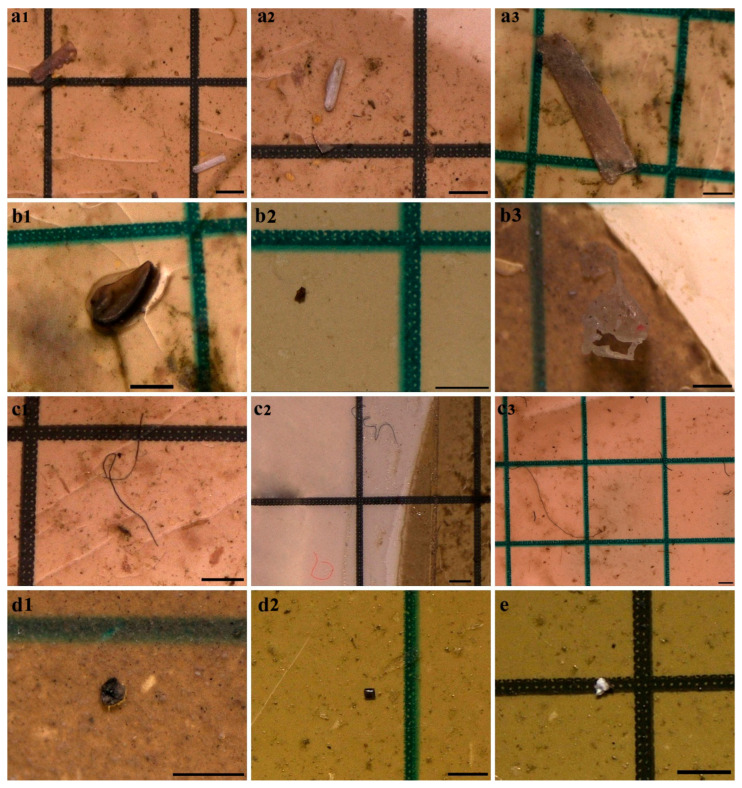
Photographs of fiber (**a1**–**a3**), fragment (**b1**–**b3**), foam (**c1**–**c3**), film (**d1** and **d2**), and pellet (**e**) microplastics observed in Greylag Goose fecal samples (Bar: 500 µm).

By particle type, fibers were dominant (n = 357; 37.22%), followed by fragments (n = 293; 30.55%), pellets (n = 180; 18.77%), films (n = 84; 8.92%), and foam (n = 45; 4.70%). Temporal variation in particle-type composition across the five composite periods is shown in Figure 4 and Figure 5 and is interpreted descriptively at the period level.

### 3.4. MP Shape

**Figure 6:**
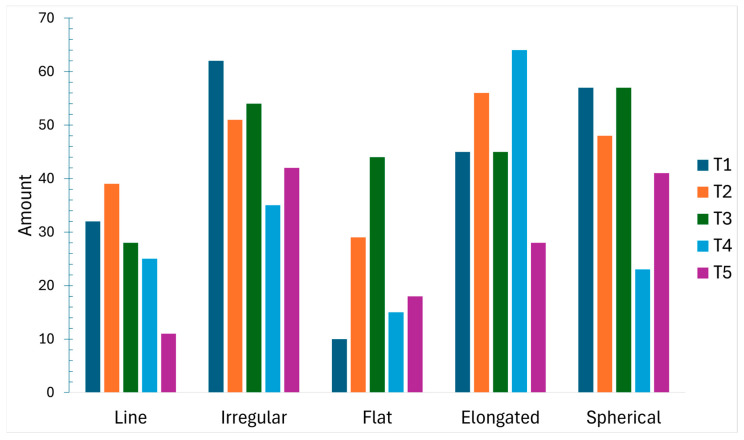
Microplastics by shape category across sampling periods (n = 5 composite samples).

MPs were categorized into five shape classes (line, irregular, flat, elongated, spherical). Irregular particles were most frequent (n = 244; 25.44%), followed by elongated (n = 238; 24.82%), spherical (n = 226; 23.57%), line (n = 135; 14.08%), and flat (n = 116; 12.09%). Temporal patterns in shape composition across the five composite periods are shown in Figure 6.

### 3.5. MP Color

**Figure 7:**
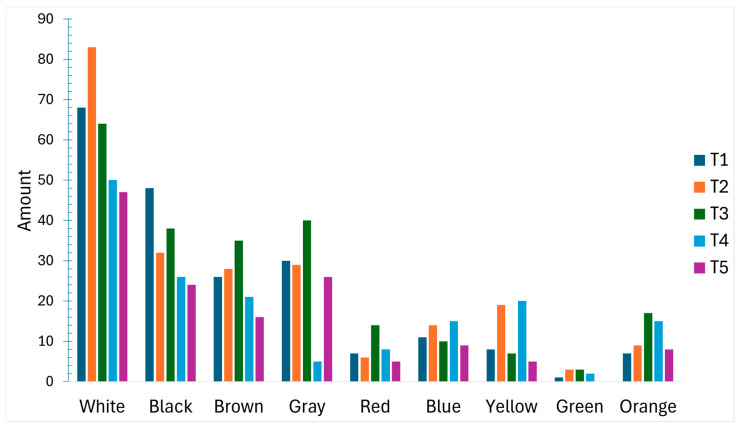
Microplastics by color category across sampling periods (n = 5 composite samples).

White/transparent particles were most frequent (n = 312; 38.52%), followed by black (n = 168; 17.52%), gray (n = 130; 13.55%), brown (n = 126; 13.14%), blue (n = 59; 6.15%), yellow (n = 59; 6.15%), orange (n = 56; 5.84%), red/pink (n = 40; 4.17%), and green (n = 9; 0.94%). Temporal patterns in color composition across the five composite periods are shown in Figure 7.

An exploratory correlation analysis (Supplementary Figure S1) suggests that fragments and pellets co-varied strongly across periods (r = 0.97), whereas fibers showed weak associations with fragments (r = 0.01) and pellets (r = −0.20) and a moderate negative association with foam (r = −0.61). Among shapes, irregular and spherical particles co-varied strongly (r = 0.94), and the largest size classes (S4–S5) were closely aligned (r = 0.92). Several color categories also co-varied (e.g., blue–yellow, r = 0.99), while others were inversely related (e.g., blue–gray, r = −0.67), indicating period-to-period shifts affecting multiple attributes concurrently.

### 3.6. Polymer and SEM Analysis

#### 3.6.1. Polymer Identification (FT-IR)

**Figure 8:**
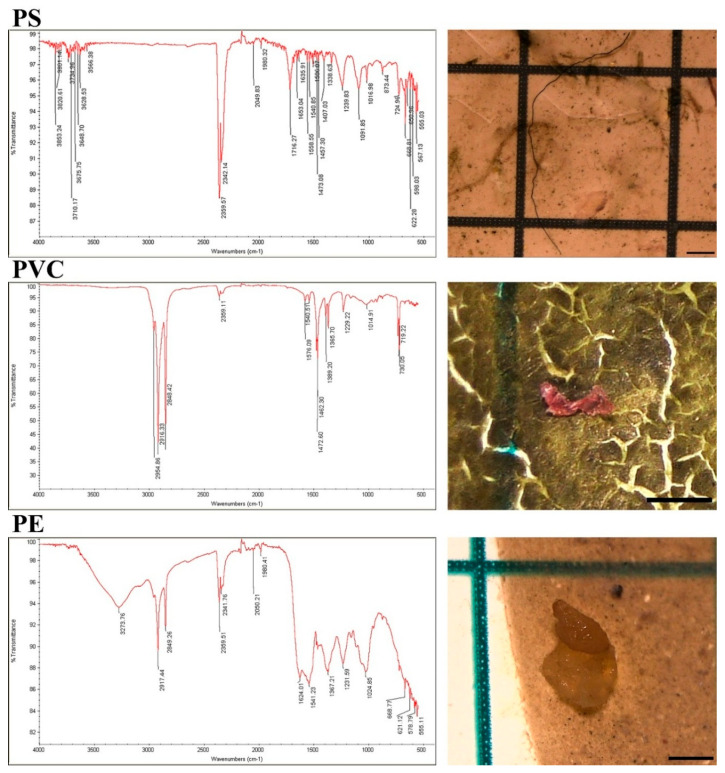
Representative ATR-FTIR spectra of identified MPs (PS, PVC, and PE). Measured spectra are overlaid with the corresponding reference/library spectra used for polymer assignment (bar: 500 µm).

FT-IR analysis of a representative subset of particles (>300 µm; ~20% of recovered items) identified three main polymers: polystyrene (PS; 44.93%), polyethylene (PE; 28.07%), and polyvinyl chloride (PVC; 6.92%). The remaining spectra were assigned to other polymers or could not be confidently matched. The predominance of PS and PE is consistent with common consumer and packaging plastics, supporting catchment- and shoreline-derived inputs into the lake system (Figure 8). In Figure 8, the measured ATR-FTIR spectra of representative particles are shown overlaid with the corresponding reference/library spectra used for polymer assignment [42]. Identification relied on matching diagnostic band positions characteristic of PS, PE, and PVC, and the observed band positions were consistent with the reference spectra. Minor differences in relative intensities and baseline shape can occur due to environmental weathering (e.g., oxidation and additives), residual surface films, and ATR contact/particle thickness effects; however, no systematic or large deviations were observed that would compromise polymer assignment.

#### 3.6.2. SEM–EDX Characterization

**Figure 9:**
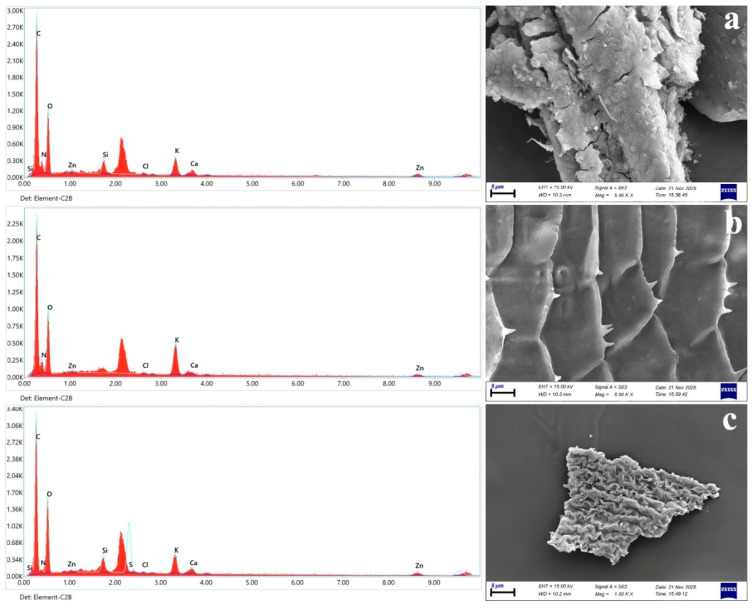
SEM images and corresponding EDX spectra of MP film (**a**), fragment (**b**), and fiber (**c**). Red-filled traces show measured X-ray counts versus energy; the blue curve shows the software-processed/fitted (background/fit) visualization used for peak assignment.

SEM imaging revealed surface abrasion, irregular textures, and cracking consistent with environmental weathering and/or mechanical stress during transport and gut passage. EDX spectra were dominated by carbon signals with additional minor elements, supporting the interpretation that the inspected particles were polymeric materials with surface-associated residues (Figure 9). In Figure 9, the red-filled spectra show the measured EDX X-ray counts as a function of energy, whereas the superimposed blue curve represents the software-processed/fitted (background/fit) visualization used during peak assignment [43] A consistent peak observed between the Si (≈1.74 keV) and S (≈2.31 keV) lines occurs in the ~2.0–2.1 keV region, where phosphorus Kα (2.013 keV) and the Au Mα line (~2.12 keV) may overlap. Because particles were sputter-coated with Au prior to SEM imaging, we attribute this feature primarily to the conductive Au coating rather than the polymer matrix itself, and we focus our interpretation on the remaining minor elemental signals associated with surface residues [44].

## 4. Discussion

Wetland ecosystems can act as both sinks and redistribution zones for microplastics (MPs) because they intercept catchment runoff, wastewater-linked inputs, and shoreline litter, thereby creating persistent exposure contexts for wildlife [45,46]. Birds are increasingly used as bioindicators of plastic contamination, and non-invasive feces-based sampling is widely regarded as an ethically robust and scalable approach for exposure surveillance [11,17,18,23,24,25,26]. Feces-based monitoring can serve as a useful proxy for waterbirds because it reflects integrated exposure via drinking water, sediment contact, and diet, while reducing biases inherent to necropsy-only datasets [23,26]. In this study, we quantified MPs in Greylag Goose (*Anser anser*) fecal deposits from Lake Erçek across five sampling periods (October–February; T1–T5). Because the Greylag Goose is a common wintering migrant at the lake, the present findings provide a first baseline for this inland wetland system in Eastern Anatolia and support the feasibility of repeated temporal monitoring in live bird assemblages [17,24].

Across five time-specific composite samples derived from 400 fecal deposits, we recovered 959 suspected MP items, corresponding to an estimated 1.75–2.85 MPs/individual (items per fecal deposit (composite-derived)), indicating measurable exposure in this population [23,24]. These per-individual magnitudes fall within the range reported by feces/guano-based studies on waterbirds and wetland-associated birds, supporting that inland systems can yield burdens comparable to those observed in protected reserves and agricultural landscapes [17,24]. More broadly, freshwater plastic pollution is widely recognized to have ecological consequences and is often under-characterized relative to marine systems, which increases the value of lake-scale baselines such as those generated here [45,46]. Seasonal (period-to-period) variability in feces- or pellet-based microplastic signals has been reported across bird systems, reinforcing that avian indicators can reflect temporally dynamic exposure fields rather than a constant background [47,48]. Studies explicitly addressing spatiotemporal patterns further show that plastic magnitude and composition associated with birds can shift across sampling periods as habitat use and foraging arenas change, thereby modulating contact with contaminated substrates and prey [48,49]. Accordingly, our late autumn–early winter peak (Time 2–Time 3) followed by a decline toward late winter (Time 5) is consistent with evidence that fecal/pellet metrics can track seasonal changes in environmental availability and bird behavior [47,50]. Because our material was pooled into one composite per period, these differences are best interpreted as period-level exposure signals, and future work should incorporate individual-level replication within each period to quantify within-period variance and strengthen inference [18,25].

The higher counts observed in Time 2–Time 3 can likely be explained by a late-autumn to early-winter convergence of catchment delivery processes and within-wetland redistribution, rather than a constant exposure regime across the season [49,51]. However, we did not have concurrent station-based rainfall (event totals) or lake-level data for Lake Erçek during the sampling periods to directly test this mechanism. Nevertheless, official long-term climate statistics for Van show that precipitation remains notable during the late-autumn/early-winter months (October–December mean monthly totals: 46.5, 47.1, and 37.5 mm; 1939–2024), indicating that runoff-generating events are plausible during the study season [52]. Regional hydrometeorological analyses in the Lake Van basin similarly highlight pronounced seasonality and variability in precipitation and related hydroclimatic parameters, supporting the expectation that material fluxes can change between months [53]. In freshwater wetland mosaics, rainfall-driven runoff and episodic high-flow events can mobilize land-based plastics, resulting in pronounced increases during discrete events rather than gradual background inputs [54,55,56,57,58,59]. Concurrently, hydrodynamic forcing and water-level fluctuations can redistribute previously deposited plastics toward shallow margins and shoals—zones that are intensively used by grazing and dabbling waterbirds—thereby increasing encounter rates even without proportional new inputs [1,51]. Temporal variability in bird-dropping-based microplastic signals has also been documented elsewhere, supporting the expectation that guano/feces loads can shift with seasonally changing habitat use and local contamination fields [27,47]. Finally, when flock size and site fidelity are high during the wintering period, repeated use of the same roosting and foraging patches can increase contact with contaminated sediments and nearshore waters, potentially reinforcing a Time 2–Time 3 signature at the period level [17,24]. This seasonal variability is consistent with wetland literature indicating that MP availability can fluctuate with hydrology and human activity, including changes in runoff delivery, water-level dynamics, and shoreline accumulation–resuspension processes [45,46]. These considerations also align with best-practice guidance that highlights how sampling structure, contamination controls, and analytical thresholds can materially influence reported MP abundance and comparability among studies [21,26].

Regarding particle morphology, recovered items were dominated by fibers (37.22%), followed by fragments (30.55%) and pellets (18.77%), indicating multiple source categories and exposure routes [17,46]. Fiber dominance aligns with numerous avian feces/guano studies in which fibers are the most frequently recovered form, often linked to textile-derived microfibers and wastewater pathways [11,17]. Similar patterns have been reported in inland waterfowl feces, supporting that thread-like materials and fibers can be prevalent in grazing-associated wetland settings [23,26]. The relatively high proportions of fragments and pellets observed here likely reflect additional contributions from packaging/consumer plastics and secondary fragmentation products, consistent with wetland studies describing heterogeneous MP mixtures under diverse land-use pressures [1,46].

The size distribution was skewed toward smaller fractions, with 100–300 µm as the most frequent class (30.25%) and <100 µm also notable (17.62%), a pattern consistent with fecal egestion favoring smaller particles that traverse the gastrointestinal tract [18,25]. Feces-based MP studies emphasize that detection limits and minimum size thresholds can drive apparent differences in abundance and composition; therefore, size-binned reporting improves interpretability across datasets [25,42,60]. Accordingly, the prominence of fine fractions in our samples strengthens the rationale for maintaining stringent contamination control and consistent reporting conventions, as recommended in best-practice frameworks for bird–plastic studies [18,25,33,42,61,62].

Color composition was dominated by white/transparent particles (38.52%), followed by black and grey. This is commonly interpreted as reflecting a mixture of weathered plastics and fibers influenced by environmental aging rather than exclusively fresh, recently released items [11,17]. SEM micrographs (Figure 9) showed irregular fragments with pronounced surface roughness, microcracks, and flaking, consistent with mechanically abraded and photo-oxidatively weathered secondary microplastics [1,63]. Such textures are widely reported for environmentally aged particles and are expected where plastics reside in shallow margins subject to repeated wetting–drying cycles, sediment contact, and physical scouring [63,64]. EDX spectra were dominated by carbon and oxygen, whereas minor signals (e.g., Si, Cl, K, Ca, Zn) were more plausibly attributable to mineral/organic surface films, sediment residues, or adsorbed constituents than to the polymer backbone itself [40,65]. Overall, SEM–EDX supports that the recovered particles represent environmentally processed plastics that likely interacted with lake sediments/biofilms prior to gastrointestinal passage and excretion [40,63].

ATR-FTIR confirmation (Figure 8) indicates that the analyzed subset includes PS, PE, and PVC, supported by diagnostic aromatic bands for PS, characteristic CH_2_ stretching/bending for PE, and PVC-associated bands linked to C–Cl containing structures [66,67]. The prominence of PS and PE is environmentally plausible because these polymers dominate packaging/consumer applications at the global scale and can generate buoyant fragments that accumulate along shorelines and shallow wetlands where waterfowl forage [1,2]. Polymer fingerprints in avian feces/scat can vary markedly across habitats and species, pointing to local waste streams and foraging niches as important determinants of exposure [27,40]. Because confirmation was restricted to a size-selected fraction (>300 µm) and a subset of particles, the relative shares of PS/PE/PVC should be treated as indicative rather than exhaustive; extending confirmation across size classes would improve comparability and reduce analytical bias [33,67]. This limitation is particularly important because the <300 µm fraction dominated the dataset, and conclusions on polymer prevalence should be interpreted accordingly.

From an ecological perspective, Greylag Goose foraging (grazing, sediment contact in shallow margins, and repeated use of wetland–agricultural mosaics) plausibly increases incidental MP ingestion via contaminated water, sediment, and diet-associated pathways [23,26]. However, fecal MPs reflect egestion and do not allow us to discriminate passive transit (e.g., ingestion with water/sediment or externally adhered particles) from deliberate ingestion of plastic items. Pairing fecal monitoring with concurrent water/sediment sampling and prey-item screening would help to resolve exposure pathways in future studies. Although we did not measure water or sediment concurrently, published studies document microplastic contamination in the wider Lake Van closed basin, including surface waters of Van Bay and multiple aquatic environments across the Van Lake drainages [30], as well as evidence from Lake Van biota [31]. These reports support the plausibility that birds foraging within the Lake Erçek–Lake Van basin are exposed via local environmental pathways. Comparable work indicates that site-level contamination and foraging behavior can influence microfiber occurrence in waterbird feces, supporting the interpretation that habitat use may mediate exposure intensity at Lake Erçek [24,26]. To strengthen source attribution and evaluate trophic transfer, future studies should pair fecal monitoring with parallel measurements in sediments, water, and potential food items, consistent with food-web evidence that MPs can occur across sediments, invertebrates, and bird-associated matrices [11,46]. Finally, growing evidence of micro(nano)plastic contamination in bird tissues highlights the broader ecological relevance of avian exposure assessment and reinforces the need for standardized, regionally representative baselines [18,21].

For spatial comparisons, the same protocol could be applied across multiple shoreline sectors representing distinct land-use pressures, using replicated composites (or preferably unpooled samples) per site and period. For interannual comparisons, repeating the same five-period winter design over multiple years would enable year-to-year contrasts while controlling for seasonality; importantly, incorporating within-period replication would allow uncertainty estimation and more robust inference.

## 5. Limitations of the Study

This study used time-specific composite samples (one composite per period), which precludes within-period replication; therefore, temporal differences are reported descriptively at the period level rather than as individual-level statistical inference. Each period-level composite, however, pooled 80 fresh deposits (n = 400 total), improving population coverage while still precluding within-period variance estimation. Because fecal deposits were collected from free-ranging flocks without individual marking, we cannot confirm that each deposit represents a unique individual; abundance is thus expressed as items per fecal deposit (composite-derived). Polymer confirmation was performed only for a subset of particles > 300 µm (~20% of recovered items), while the dominant < 300 µm fraction was not chemically validated; accordingly, polymer composition should be considered indicative. Finally, although basic contamination-reduction steps were implemented, quantitative field and procedural blanks were not performed; thus, we could not correct for airborne contamination (particularly fibers), which may inflate fiber counts. Future work should incorporate systematic blanks and broader spectroscopic confirmation across size classes (e.g., micro-FTIR or micro-Raman mapping for <300 µm particles) following recent methodological recommendations for biological matrices [41].

## 6. Conclusions

This study provides the first baseline evidence of microplastic exposure in Greylag Goose feces from Lake Erçek, an inland soda lake in Eastern Anatolia. MPs were detected in all sampling periods, with period-level abundance peaking in late autumn-early winter and declining toward late winter. Particle composition was dominated by fibers and small size fractions, and FT-IR confirmed common consumer polymers (PS, PE, PVC) in a representative subset. The results demonstrate that feces-based monitoring can be applied effectively to inland wetland birds in Türkiye and can support future, standardized surveillance integrating environmental matrices for improved source attribution and risk evaluation.

## Figures and Tables

**Table 1:** Data from *Anser anser* fecal samples.

Point	Number of Samples	Number of Homogenized Samples	MP Particles
Time 1	80	1	206
Time 2	80	1	223
Time 3	80	1	228
Time 4	80	1	162
Time 5	80	1	140
**Total**	**400**	**5**	**959**

## Data Availability

The original contributions presented in this study are included in the article/Supplementary Materials. Further inquiries can be directed to the corresponding author.

## Supplementary Materials

The following supporting information can be downloaded at: https://www.mdpi.com/article/10.3390/toxics14020108/s1, Table S1: Descriptive statistics, Figure S1: Correlation matrices among period-level MP attributes; Table S2: ANOVA results; Table S3: Duncan’s multiple range test results (provided for reference, if needed).
