# Supplementary figures and images for: Microplastics in Greylag Goose (Anser anser) Feces from Lake Erçek (Eastern Anatolia, Türkiye): Occurrence, Temporal Variation, and Polymer Characterization
Source: Toxics. 2026 Jan 23;14(2):108. doi: 10.3390/toxics14020108 (PMC12944368; doi:10.3390/toxics14020108)

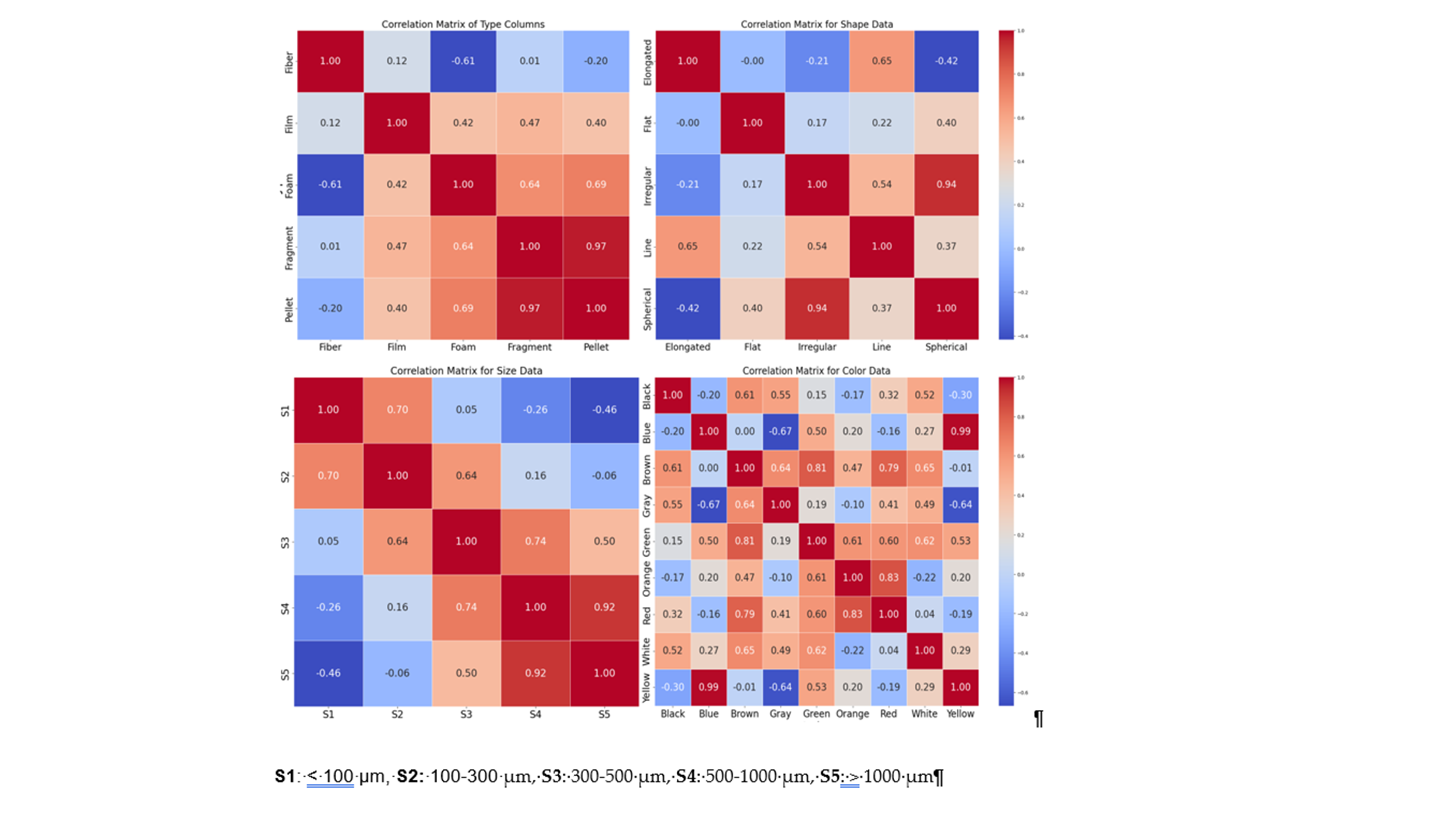

Supplement: Supplementary file 1 [file toxics-14-00108-s001.zip › Figure S1.png]
